# Augmented and Virtual Reality for Preoperative Trauma Planning, Focusing on Orbital Reconstructions: A Systematic Review

**DOI:** 10.3390/jcm12165203

**Published:** 2023-08-10

**Authors:** Kathia Dubron, Maarten Verbist, Reinhilde Jacobs, Raphael Olszewski, Eman Shaheen, Robin Willaert

**Affiliations:** 1 Department of Oral and Maxillofacial Surgery, University Hospitals Leuven, 3000 Leuven, Belgium; 2 OMFS IMPATH Research Group, Department of Imaging & Pathology, University Hospitals Leuven, 3000 Leuven, Belgium; reinhilde.jacobs@ki.se; 3 Department of Oral and Maxillofacial Surgery, Cliniques Universitaires Saint Luc, UCLouvain, Av. Hippocrate 10, 1200 Brussels, Belgium; 4 Department of Dental Medicine, Karolinska Institutet, 171 77 Stockholm, Sweden; 5 Oral and Maxillofacial Surgery Research Lab (OMFS Lab), NMSK, Institut de Recherche Expérimentale et Clinique (IREC), SSS, UCLouvain, 1200 Brussels, Belgium

**Keywords:** traumatology, orbital fractures, orbital implants, preoperative planning, virtual reality, augmented reality, computer-assisted image analysis

## Abstract

Background: This systematic review summarizes recent literature on the use of extended reality, including augmented reality (AR), mixed reality (MR), and virtual reality (VR), in preoperative planning for orbital fractures. Methods: A systematic search was conducted in PubMed, Embase, Web of Science and Cochrane on 6 April 2023. The included studies compared extended reality with conventional planning techniques, focusing on computer-aided surgical simulation based on Computed Tomography data, patient-specific implants (PSIs), fracture reconstruction of the orbital complex, and the use of extended reality. Outcomes analyzed were technical accuracy, planning time, operative time, complications, total cost, and educational benefits. Results: A total of 6381 articles were identified. Four articles discussed the educational use of VR, while one clinical prospective study examined AR for assisting orbital fracture management. Conclusion: AR was demonstrated to ameliorate the accuracy and precision of the incision and enable the better identification of deep anatomical tissues in real time. Consequently, intraoperative imaging enhancement helps to guide the orientation of the orbital reconstruction plate and better visualize the precise positioning and fixation of the PSI of the fractured orbital walls. However, the technical accuracy of 2–3 mm should be considered. VR-based educational tools provided better visualization and understanding of craniofacial trauma compared to conventional 2- or 3-dimensional images.

## 1. Introduction

Orbital trauma is a prevalent injury of the maxillofacial complex, and its three-dimensional (3D) anatomy can necessitate challenging reconstructions [1]. Current surgical procedures for orbital reconstruction focus mainly on the reconstruction of the bony anatomy and aim to restore the original orbital volume to achieve a functional and aesthetically pleasing outcome [2,3,4]. However, precise repositioning or reconstruction of displaced fracture parts can be challenging because of the lack of direct visualization during the operation. New technologies such as virtual surgical planning, virtual reality (VR), augmented reality (AR), patient-specific implants (PSIs), and intraoperative navigation can enhance optimal complex orbital reconstructions [5,6]. Virtual reality (VR) refers to the virtual environment created to assess the maxillofacial anatomy for the diagnosis, planning and surgical simulation of these complex anatomical regions [7]. VR can be either immersive or non-immersive. Immersive VR uses a wearable device to create a sense of interaction with the virtual environment. In non-immersive VR, virtual experiences are generated on a desktop. This concept is also known as virtual planning or computer-assisted surgical simulation (CASS). AR means superimposing a patient-specific 3D environment onto the real world, for example, the operative field [8]. In this case, a visualization device such as a head-mounted device is necessary to augment the virtual scene [7]. Lastly, mixed reality (MR) is a hybrid technology combining AR and VR. However, the two terms MR and AR can also be considered to have the same meaning [9]. Real and virtual images can be manipulated in the real and virtual worlds. Intraoperatively, a surgeon can access anatomical information and overlay virtual holographic elements, and bidirectional communication with colleagues is possible [10]. To conclude, virtual 3D images can be precisely planned and manipulated preoperatively. These images can be used during surgery in the context of AR, VR, or MR, enabling optimal orbital reconstruction and reducing operative time [11]. In the present literature, a PSI is postulated as beneficial because of its precise reconstruction of the orbital floor by using a digital workflow and is considered superior to manually bent “patient adapted” titanium mesh implants [12].

The main objectives of these technologies are to enhance precision and consistency, leading to reductions in planning time, surgical duration, complications, and overall intervention costs. Additionally, there is a growing interest among medical trainees and experienced surgeons to integrate virtual reality into OMFS training programs and thus further improving technical accuracy [13]. Considering that AR, VR, and MR can potentially increase the precision of positioning a PSI for orbital reconstruction, this systematic review aimed to provide an overview of the state of the art of extended reality and offer further insights for advancing this technology for clinical practice and educational purposes.

## 2. Materials and Methods

This systematic review compared the use of extended reality in preoperative planning for orbital trauma as part of conventional planning methods. The review protocol was prospectively registered with PROSPERO (registration number: CRD42020218150). The Preferred Reporting Items for Systematic Reviews and Meta-Analysis (PRISMA) guidelines were followed during the literature search and writing [14]. All studies included in this systematic review met the criteria established by the PICOS approach (patient population, intervention, comparison, outcomes, and study design).

A systematic search string was conducted on 6 April 2023 on the following databases: PubMed (Medline), Embase, Web of Science (Core Collection), and Cochrane. For each database, a constructed search string was used to identify studies that included two concepts: orbital fractures and extended reality for virtual preoperative planning or intraoperative use. The search strategies for all databases can be found in Appendix A.

The articles found were checked for duplicates, and their titles and abstracts were scanned for eligibility. Disagreements between the two reviewers were resolved by a third reviewer.

The inclusion criteria for this review were the following: (I) preoperative virtual planning based on CT scan with the use of extended reality (VR, MR or AR); (II) patients needed to have an orbital blow-out fracture (floor, medial or lateral orbital wall fractures) or an orbitozygomatic fracture needing surgical management; (III) preclinical studies on human specimens or printed skulls; and (IV) studies about the educational impact of VR, AR and MR. The exclusion criteria involved case reports (<2 cases), animal studies, opinion-based research, pediatric population (<18 years), outdated research (<1992), isolated zygomatic arc fractures, epidemiological research and non-surgical management. Articles in languages other than English were excluded.

After the article selection, the quality was evaluated by two independent authors using the MINORS assessment scale [15]. Non-comparative studies were graded from 0 to 16 (the global ideal score) for eight items, and comparative studies were graded from 0 to 24 (the global ideal score) for twelve items. All MINOR items were individually scored 0 (not reported), 1 (reported but inadequate) or 2 (reported and adequate). Meta-analysis was not possible due to the lack of quantitatively comparable data among the databases. The quality assessment is shown in Table 1.

## 3. Results

**Figure 1:**
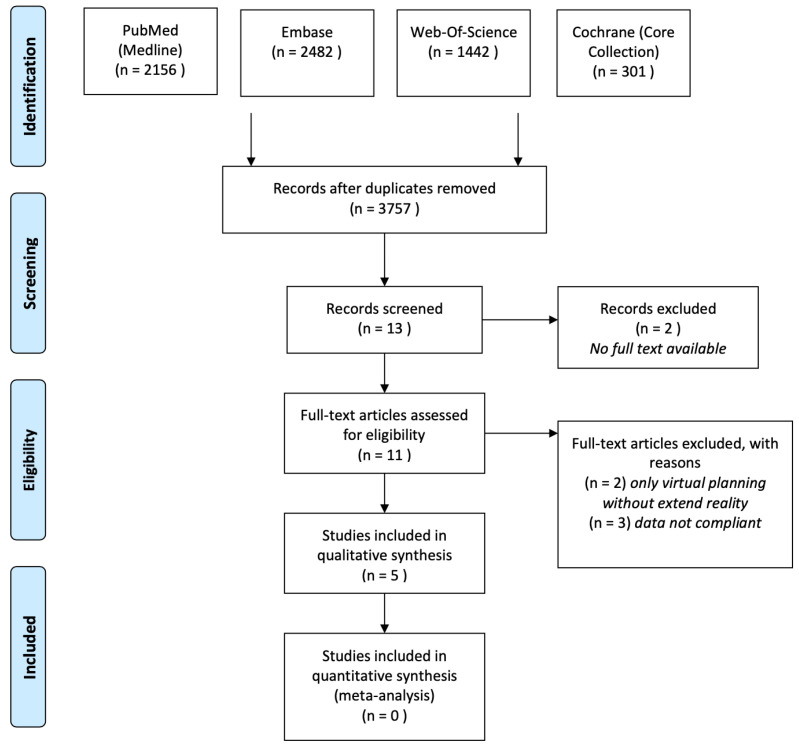
Flowchart (Prisma 2009) of article selection.

A total of 6381 articles were identified by reviewing the medical databases mentioned above. After removing duplicates and screening the titles and abstracts, eleven articles were selected (Figure 1), of which five full-text articles were identified for eligibility. Table 1 provides a detailed overview of the patient demographics and the study characteristics of the five included studies.

### 3.1. Preoperative Planning Time

Preoperative planning time was longer compared to the conventional planning method. Two to five days were needed to make the preoperative virtual plan (Table 2) [17].

### 3.2. Operative Time

This ranged from 60 to 135 min, averaging 103 min (Table 2) [17].

### 3.3. Technical Accuracy and Outcome

The average distance between the postoperative implant position and the virtually planned position was 0.432 mm. Three months postoperatively, this average distance increases slightly to 0.496 mm (Table 2) [17].

Rahimov et al. showed a mean deviation of 0.9 mm, ranging from 0.65 to 1.15 mm [20]. However, this study was performed using Microsoft HoloLens-printed skulls. In two cases, the surgeon was not able to concentrate their attention on both holographic and real objects [20]. Another study [19] calculated the technical accuracy of a Microsoft HoloLens on printed fractured orbits by calculating fiducial registration errors, target registration errors and guidance errors. A mean error of 2–3 (±1) mm was observed for all three errors. This error, however, was user-dependent and based on an experiment with three participants (one surgeon).

### 3.4. Complications

None of the patients showed any type of complication (Table 2).

### 3.5. Satisfaction Score

Patients treated with AR indicated a high satisfaction rate, ranging from 8 to 10 on a 0 to 10 scale, with an average satisfaction score of 9.4 points [17]. Furthermore, high satisfaction scores were found for the trainees who used a VR environment to provide diagnoses and treatment plans (Table 2) [18].

It was reported that AR provides valuable data to the surgeon without requiring significant changes or adaptations of the surgical technique, and it gives immediate feedback to the surgeon [16]. Also, the system is ergonomically friendly [16].

### 3.6. Education

Experts using a cognitive VR simulator called Touch Surgery outperformed novices in the orbital floor reconstruction module [19]. Additionally, the VR environment DIVA (Data Integration and Visualization in Augmented and Virtual Environments, Pasteur Institute, Paris) offers trainees a better understanding and a more intuitive approach to craniofacial trauma. Moreover, DIVA allows the precise visualization of distinct lesions not visible in the usual 2D and 3D images (Table 2) [18]. Mixed reality with a head-mounted device and practicing the surgery beforehand could significantly improve the surgical training and, therefore, the outcome of the surgery [8].

## 4. Discussion

In this study, the workflow of preoperative planning in orbital reconstructions with the use of for AR/VR technology will be discussed. The current process involves acquiring volumetric data from medical imaging, converting it to the digital imaging and communication in medicine (DICOM) format, performing segmentation, and generating 3D renderings for virtual surgical planning and designing a PSI. During the surgical procedure and preoperative evaluation, either 3D models or AR/VR technologies can be used [21,22].

Virtual planning of a PSI for orbital reconstruction requires virtual restoration of the orbital walls based on the segmentation of CT images. However, orbital walls are complicated structures, and their low thickness and rendering can cause artificial defects. This may lead to a misrepresentation of conventional CT. Recent studies have investigated automated virtual reconstructions and have observed that in certain clinical cases, automated segmentation could yield comparable clinical outcomes while concurrently reducing planning time [23].

VR and AR are fast-developing technologies that allow the real-time (dynamic) 3D visualization of digital information in a virtual environment [16,17]. Moreover, AR is gaining in popularity since it provides an immersive information interface for the patient with see-through visual cues [19]. Therefore, AR can be beneficial in clinical applications, whereas VR can be more useful for educational purposes. In the present literature, however, clinical information about the implementation of AR is scarce and based mainly on phantom or cadaveric studies [24]. This systematic review aimed to analyze current insights to advance this technology for clinical practice and education.

Chen et al. documented the several advantages of AR in unilateral orbitozygomatic complex fracture treatment [17]. Firstly, it improves the preoperative explanation of the surgical procedure. It also helps the surgeon plan the length and direction of the incision more accurately and on an individual basis. AR helps identify the deep anatomical tissue and guides the surgeon in real time. It provides a reference for the shape of the orbit by projecting the height, width, and radian of the virtually planned reduction on the operation field. Lastly, it gives immediate feedback about the desired implant position. Chen et al. acknowledged that the basic technology is still not perfect for orbital reconstruction, posing problems such as the complexity of tracking and the impact of direct light on accuracy. Although only a few patients have been treated with this technique, AR has additional benefits such as strengthening the preoperative communication, facilitating the surgical plan, reducing the operation time, providing guidance during the operation, and improving the accuracy of surgery compared to the traditional method. Nevertheless, preoperative planning may be lengthened; it results in time savings during the intraoperative phase. Regrettably, no study has investigated the distinct stages involved in the planning process, making it unclear to provide precise data to identify the source of the delay.

Two AR applications have recently been approved for neurosurgery (Brainlab, Munich, Germany) and spine surgery (Augmedics X-vision, Arlington Heights, IL, USA). There is no system for craniofacial surgery approved by the Food and Drug Administration (FDA) [16]. However, there are in vitro experiments using Microsoft HoloLens for orbital surgery. The aim is to provide the surgeon with immediate feedback about the position of the implant according to three axes (*x*, *y*, and *z*). Maximal accuracy is obtained when the Implant position corresponds completely to the preoperatively planned position, in which case the error should be 0 mm. However, a mean error of 2 to 3 mm is observed with a standard deviation of 1 mm [19]. Given the small operation area and the high aesthetic and functional demands of an accurately placed orbital implant, this error could be problematic. AR systems used in other surgical disciplines also showed a mean error of 2.9 mm [8]. Possible reasons for this inaccuracy include crude hardware, the tracking algorithm, the motion of the AR marker during algorithm processing, and calibration errors [16]. Despite the problem of inaccuracy, surgeons are satisfied with the AR–surgical workflow [16].

Lastly, on an educational level, presurgical VR training can increase confidence during operations and reduce overall operating times and surgical errors made by trainees [25]. To verify this statement in the OMFS field, Bouaoud et al. researched the use of VR in training with 50 students using a VR platform (DIVA) in craniofacial trauma (including orbital trauma) [18]. Of the total, 92% of students reported a high degree of satisfaction in analyzing craniofacial trauma cases. They were able to visualize lesions that would be invisible on 2D and 3D models. The mean 3D errors for positioning 3D landmarks were lower than 1 mm, ranging from 0.37 to 0.84 mm. The authors conclude that a VR learning platform such as DIVA can provide more insights into the understanding of complex orbital fractures, complementing conventional teaching methods.

However, reconstruction of the orbital floor still necessitates the retraction of the eyeball and deep dissection through a small incision to safely place a patient-specific implant beneath the eye. In general, the precise positioning of the implant depends mostly on the surgeon’s expertise. Nowadays, virtual planning and intraoperative imaging or navigation are new tools to optimize the precision of implant positioning [6,25,26]. In the future, extended reality may play a role in intraoperative positioning, optimize accuracy, and reduce operative costs. Its integration into clinical practice offers an opportunity to gain more clarity on associated financial aspects, thereby warranting further research in healthcare settings. Additionally, more research is needed, and both FDA and Medical Device Regulation approval are required. Furthermore, restoring the bony architecture of the orbit is only one part of the reconstruction. Soft tissues occupying the orbital cavity, such as fat and muscles, also play an important role in determining the orbit and, more specifically, the globe position. Both hard and soft tissues should be considered when planning orbital reconstruction [24,27]. Unfortunately, no consensus exists yet on how to integrate soft tissue measurements during virtual treatment planning. Nevertheless, new techniques of globe positioning and soft tissue volumetry could add valuable information to establish a more reliable reconstructive algorithm [27].

## 5. Conclusions

The use of AR/VR technology in the medical field is a rapidly evolving technology that holds great potential for improving preoperative planning for orbital trauma reconstruction. The current study, one of the first to focus on this application of AR/VR technology, added to the growing body of knowledge in this field and highlighted the importance of incorporating these technologies in clinical practice.

As shown by this systematic review, AR/VR technologies have the ability to assist surgeons in planning the length and direction of incisions more accurately. AR can help identify the deep anatomical tissues in real time, thus providing a reference for the shape of the orbit and projecting the height, width, and radial height of the virtually planned fracture reduction.

Additionally, there is significant interest from medical trainees and students due to the better understanding of craniofacial trauma patterns offered by the new technologies and their ability to improve training in surgical decision-making skills preoperatively. It is believed that AR/VR technology will continue to play a critical role in future surgical planning, outcomes, and education.

## Figures and Tables

**Table 1:** Study characteristics and quality assessment.

Author	Study Design	Type of Fracture	Population Size	Male/Female Ratio	Mean Age (y) and SD (±) or Range	VR	MR	AR	Quality Assessment (MINORS)
Khelemsky et al., 2016 [16].	Cross-sectional study	Orbital floor	39 students, 10 experienced surgeons	18/20 (students) 9/1 (experiences surgeons)	24.8 ± 2.5 46.6 ± 9.8	Touch Surgery (Kinosis Ltd., London, UK)	No	No	13/24
Chen et al., 2020 [17].	Prospective case series	Unilateral orbitozygomatic maxillary complex	9 patients	5/4	39.1	Intomimics 16.0 software	(Yes)	HuaxiAR1.0 software system	11/16
Bouaoud et al., 2021 [18].	Cross-sectional study	Craniofacial trauma, including orbital floor	50 students, 4 postgraduate students	/	/	DIVA (Data Integration and Visualization in Augmented and Virtual Environments, Pasteur Institute, Paris)	(Yes)	DIVA (Data Integration and Visualization in Augmented and Virtual Environments, Pasteur Institute, Paris)	6/16
Liu et al., 2021 [19].	Experimental prospective	Orbital fracture, 3D-printed skull	1 surgeon	/	/	Microsoft Hololens (Microsoft, Redmond, WA, USA)	No	Yes	8/16
Rahimov et al., 2022 [20].	Experimental prospective	Orbital fracture, 3D-printed skull	10 residents 5 experienced surgeons	/	/	Microsoft Hololens (Microsoft, Redmond, WA, USA)	Yes	No	5/16

**Table 2:** Technical accuracy of patient-specific implants to conventional method.

Author	Preoperative Planning Time	Operation Time	Technical Accuracy and Outcome	Complications	Satisfaction Score	Total Cost
Khelemsky et al., 2016 [16].	/	/	Experts outperformed novices of the orbital floor reconstruction module (*p* < 0.001)	/	/	No info
Chen et al., 2020 [17].	2–5 days	103.3 min (mean time), 60–135 min (range)	Postoperative vs. the virtual surgery plan: between 1.5–2 mm * Injured side vs. healthy side of the face on preoperative: ±0.5 mm * Injured side vs. healthy side of the face 3 months postoperative: ±0.5 mm *	None	9.4/10 (mean), 8/10–10/10 (range) **	No info
Bouaoud et al., 2021 [18].	/	/	Better understanding of craniofacial trauma using virtual reality (98%) Easy and intuitive (88%) Visualization of some lesions not seen on the usual 2D and 3D renderings (82%) Distances between trials (morphometric analysis) (*p* > 0.05) Variability in anatomical landmark positioning <1 mm	/	98% ***	No info
Liu et al., 2021 [19].	/	/	Mean error: 2–3 (±1) mm	/	Good	No info
Rahimov et al., 2022 [20].	/	/	0.9 mm (0.65–1.15) (linear accuracy) maximum: 1.75 mm	/	/	Increased

* = mean distance error, ** = patients, *** = students/participants.

## Data Availability

Not applicable.

## Appendix A

Full search string:

**Database**	**#Refs**	**#Refs after Deduplication**
PubMed (Medline)	2156	
Embase	2482	
Web of Science	1442	
Cochrane (Core Collection)	301	
*Total*	6381	3757

PubMed (Medline)
(“orbital fractures”[Mesh] OR ((“orbit surgery”[tiab] OR orbit*[tiab] OR blow-out*[tiab] OR “orbitozygoma*”[tiab] OR orbital-wall[tiab] OR orbital-floor[tiab]) AND (“Fractures, Bone”[Mesh:NoExp] OR fracture*[tiab]))) AND (“orbital implants”[Mesh] OR orbital-implant*[tiab] OR patient-specific-implant*[tiab] OR “Imaging, Three-Dimensional”[Mesh] OR 3D[tiab] OR three-Dimensional[tiab] OR 3-D[tiab] OR “Virtual Reality”[Mesh] OR virtual*[tiab] OR augmented-realit*[tiab] OR mixed-realit*[tiab] OR “Reconstructive Surgical Procedures”[Mesh:NoExp] OR reconstructive-surg*[tiab] OR computer-assisted[tiab] OR computer-aided[tiab] OR navigation*[tiab] OR management[tiab] OR preoperative-plan*[tiab] OR planning*[tiab] OR pre-operative-plan*[tiab] OR pre-surg*[tiab] OR presurg*[tiab] OR pre-plan*[tiab] OR preplan*[tiab] OR preoperative-care[tiab] OR preparation*[tiab])
Embase
(‘orbital fractures’/exp OR ((‘orbit surg*’:ti,ab,kw OR ‘orbit*’:ti,ab,kw OR ‘blow out*’:ti,ab,kw OR ‘orbitozygoma*’:ti,ab,kw OR ‘orbital wall’:ti,ab,kw OR ‘orbital floor’:ti,ab,kw) AND ‘fracture*’:ti,ab,kw)) AND (‘orbital implants’/exp OR ‘orbital implant*’:ti,ab,kw OR ‘patient specific implant’/exp OR ‘three-dimensional imaging’/exp OR ‘three dimensional’:ti,ab,kw OR ‘3d’:ti,ab,kw OR ‘virtual reality’/exp OR ‘virtual*’:ti,ab,kw OR ‘augmented reality’/exp OR ‘augmented realit*’:ti,ab,kw OR ‘mixed realit*’:ti,ab,kw OR ‘reconstructive surgery’/exp OR ‘reconstructive surg*’:ti,ab,kw OR ‘computer assisted’:ti,ab,kw OR ‘computer aided’:ti,ab,kw OR navigation*:ti,ab,kw OR management:ti,ab,kw OR ‘preoperative plan*’:ti,ab,kw OR ‘planning*’:ti,ab,kw OR ‘pre operative plan*’:ti,ab,kw OR ‘pre surg*’:ti,ab,kw OR ‘presurg*’:ti,ab,kw OR ‘pre plan*’:ti,ab,kw OR ‘preplan*’:ti,ab,kw OR ‘preoperative care’:ti,ab,kw OR ‘preparation*’:ti,ab,kw)
Web Of Science
(TS=(“orbital fractures*” OR ((“orbit surgery” OR “orbit*” OR “orbitozygoma*” OR “blow out” or “orbital wall” or “orbital floor”) NEAR/6 “Fracture*”))) AND (TS=(“orbital implant*” OR “patient specific implant” OR “three dimensional” OR “3D” OR “3 D” OR “virtual*” OR “augmented realit*” OR “mixed realit*” OR “reconstructive surg*” OR “computer assisted” OR “computer aided” OR navigation* OR management OR “preoperative plan*” OR “planning*” OR “pre operative plan*” OR “pre surg*” OR “presurg*” OR “pre plan*” OR “preplan*” OR “preoperative care” OR “preparation*”))
Cochrane (Core Collection)
((([mh “orbital fractures”] OR ([mh “orbital”] AND [mh ^”Fractures, Bone”])) OR ((blow-out* OR orbital* OR ((“orbit surgery” OR orbit* OR orbitozygoma* OR “blow out” OR “orbital wall” OR “orbital floor”) AND fracture*)):ti,ab,kw))) AND (((([mh “orbital implants” ] OR (patient specific implant*) OR [mh “Imaging, Three-Dimensional”] OR [mh “Virtual Reality”] OR [mh ^”Reconstructive Surgical Procedures”])) OR ((3D OR “three Dimensional” OR “3 D” OR virtual* OR (augmented NEXT realit*) OR (mixed NEXT realit*) OR (reconstructive NEXT surg*) OR “computer assisted” OR “computer aided” OR navigation* OR management OR (preoperative NEXT plan*) OR planning* OR (pre NEXT operative NEXT plan*) OR (pre NEXT surg*) OR presurg* OR (pre NEXT plan*) OR preplan* OR “preoperative care” OR preparation*):ti,ab,kw)))
